# Ecological Assets and Academic Procrastination among Adolescents: The Mediating Role of Commitment to Learning

**DOI:** 10.3389/fpsyg.2017.01971

**Published:** 2017-11-16

**Authors:** Bin-Bin Chen, Wen Han

**Affiliations:** Department of Psychology, Fudan University, Shanghai, China

**Keywords:** ecological assets, academic procrastination, commitment to learning, adolescents, China

## Abstract

Academic procrastination is defined as a purposive delay of academic tasks that must be completed. Within the framework of the ecological model of resiliency, this study examined how ecological assets were related to academic procrastination among adolescents. Participants in the study were 577 adolescents (53.5% boys) from Shanghai, China. They completed measures of ecological assets, commitment to learning, and academic procrastination. Structural equation modeling revealed that, as predicted, ecological assets were negatively associated with academic procrastination. In addition, commitment to learning fully mediated the association between ecological assets and academic procrastination. Implications of the present findings are discussed.

## Introduction

Procrastination was defined as the tendency to delay or postpone an intended course of action (Ferrari, 2001; Steel, 2007). It is highly prevalent among adolescents, and has been identified in the diverse cultural contexts (Klassen et al., 2009; Svartdal et al., 2016). As one of important domains of procrastination (Owens and Newbegin, 2000), academic procrastination is defined as a purposive delay of academic tasks that must be completed (Schraw et al., 2007). It is one of most prevalent procrastination behaviors among adolescents. It has become a particularly important issue because academic procrastination was found to be associated with poor psychological outcomes and school adjustment (e.g., Tice and Baumeister, 1997; Howell et al., 2006; Kim and Seo, 2015; Chen and Chang, 2016).

In an effort to better understand academic procrastination, a number of empirical studies have explored intra-personal factors such as personality (Johnson and Bloom, 1995; Beck et al., 2000), self-esteem (Chen et al., 2016), self-efficacy (Klassen et al., 2008), achievement motivation (Saddler and Buley, 1999), and time perspective (Chen and Chang, 2016; Chen and Kruger, 2017), which may influence academic procrastination. Despite this progress, there are relatively few studies looking at environmental factors that may be associated with procrastination including academic procrastination (Chen et al., 2016; Chen and Kruger, 2017; Nordby et al., 2017). The present study aimed to examine the associations between ecological assets and academic procrastination and the potential mediating role on the association between ecological assets and academic procrastination within the theoretical framework of an ecological model of resiliency.

The ecological model of resiliency (Furlong et al., 2009; Shekhtmeyster et al., 2011), integrating resiliency theory with ecological theory, elucidates that adolescents who have high levels of personal and ecological assets (i.e., strengths or enhancement factors) may develop resilience traits that may promote adaptive academic, social, and behavioral performances. According to this model, ecological assets which reflect adolescents’ positive developmental experiences (e.g., support from parents, security in community, participation in youth programs) have advantage to meet adolescents’ basic developmental needs, which then contribute to positive developmental outcomes among adolescents (Shekhtmeyster et al., 2011). This is because ecological assets may consistently discourage negative or maladaptive behavior and promote positive or adaptive behaviors. This model has been applied in examining adolescents’ risk-taking behaviors such as substance use. For example, previous research has shown that adolescents who had high levels of ecological assets reported less substance abuse (Shekhtmeyster et al., 2011).

Following the same logic, the present study hypothesized that ecological assets, the construct derived from the positive youth development (PYD) model, would be negatively associated with academic procrastination. The PYD model emphasizes the strengths of adolescents and their positive qualities (Lerner et al., 2009), which are considered as core aspects of youth development that were ignored by scholars who focused only on the problems of adolescents. Within the theoretical framework of the PYD model, Leffert et al. (1998) and Benson (2003, 2007) argued that adolescents should have developmental assets to promote healthy life. They proposed four categories of what they theorized as constituting ecological assets: support, empowerment, boundaries and expectations, and constructive use of time. These ecological assets reflected adolescents’ positive developmental experiences at the externally ecological level. It has become increasingly clear that ecological assets may reduce the likelihood of negative developmental outcomes and raise the likelihood of positive developmental outcomes (Benson, 2007; Li et al., 2010).

There is limited literature examining the associations between ecological assets and academic procrastination in the PYD perspective. However, early support for this hypothesis has been found in other areas. For example, a growing body of literature has indicated that the more assets adolescents reported having, the more they reported better academic achievement (Scales et al., 2006), and the less they reported to have risk behaviors and depression (Theokas and Lerner, 2006). In addition, there is complementary research that has examined some of the ecological assets categories relevant to the PYD model. For example, positive social support networks were negatively associated with general procrastination (Ferrari et al., 1998). As another example, parental and school provisions of boundaries and expectations were associated with less general procrastination (Chen, unpublished). Of particular relevance to the present study is the very limited investigation of the association between ecological assets and academic procrastination. Indeed, only a small number of studies indicated that socio-personal factors (e.g., parents’ education and number of siblings) were related to academic procrastination (Rosário et al., 2009). Although previous work did not focus mainly on procrastination in academic domain, it would be possible that ecological assets which were associated with general procrastination would be similarly associated with academic procrastination.

In addition, within the theoretical framework of the ecological model of resiliency, recent literature has suggested that ecological assets affect developmental outcomes through the mediation of intrapersonal assets. For example, ecological assets positively predicted self-esteem and perceived school importance, which, in turn, influenced psychological symptoms and antisocial behaviors (Christens and Peterson, 2011). Of particular relevance to the present study is the very limited investigation of intrapersonal asset which may be considered as a potential mediator between ecological assets and academic procrastination. One intrapersonal asset may be commitment to learning which is defined as a desire to the involvement in academic activities (Benson, 2003). A few published studies have shown the bivariate correlations between ecological assets and commitment to learning, and between commitment to learning and academic procrastination. On the one hand, literature has shown that ecological assets were positively associated with commitment to learning (e.g., Taylor et al., 2004). On the other hand, research has indicated that commitment to learning was negatively associated with academic procrastination (e.g., Rosário et al., 2009; Chen, unpublished). The present study, based on the extant literature, proposes that commitment to learning may act as a mediator in the relationship between ecological assets and academic procrastination.

The goal of this study was to test the associations among ecological assets, commitment to learning, and academic procrastination in a sample of Chinese adolescents. There were two hypotheses to be tested in the present study. First, consistent with previous research, ecological assets were hypothesized to be negatively related to academic procrastination. Second, commitment to learning was hypothesized to be a mediator in the relationship between ecological assets and academic procrastination.

## Materials and Methods

### Participants

This study utilized part of the data collected in a Chinese sub-project, initiated by the PYD cross-national project (Wiium and Dimitrova, 2015). This survey was conducted in October, 2015. We invited 600 students from five public high schools in Shanghai, People’s Republic of China. Finally, a total of 577 participants (53.5% boys) took part in the current study. It represented a 96.17% response rate. Participants ranged in age from 13 to 16 years, with a mean age of 14.56 years (*SD* = 1.05). Fathers’ and mothers’ educational levels were as follows: 18.07 and 25.22% had junior high school or lower education; 43.86 and 39.58% had a senior high school education; and 38.07 and 35.20% had at least some college or higher education.

### Procedures

Upon receiving the school authority’s permission and written consent from participants, this survey was conducted in individual classrooms. The students completed self-report measures as part of a larger research project. All students were voluntarily recruited in this project after having the purpose of the research explained to them, and were informed that they could decline to participate at any time. They were assured of their confidentiality and anonymity. They were allotted a single class period to complete the measures. It took approximately 30 min to complete the questionnaires. The administration of all measures was carried out by a group of research assistants. No teachers were present during the survey in class.

### Measures

#### External Assets Scale

Ecological assets were measured by the External Assets Scale in the Profiles of Student Life: Attitudes and Behavior survey, developed by the Search Institute (Benson, 2003). It includes four subscales including support (e.g., “I have a family that gives me love and support”), empowerment (e.g., “I am given useful roles and responsibilities”), boundaries and expectations (e.g., “I have a family that knows where I am and what I am doing”), and constructive use of time (e.g., “I am involved in a church, mosque, or other religious group one or more hours every week”). They had adequate reliability, and satisfactory content validity, construct validity, and predictive validity (Leffert et al., 1998; Scales, 1999). Items were presented on a four-point scale ranging from 1 = “rarely” to 4 = “almost always.” It proved satisfactory reliability and validity in previous Chinese literature (Chen et al., 2017). Internal consistency reliability estimates were 0.79 for support, 0.82 for empowerment, 0.89 for boundaries and expectations, and 0.73 for constructive use of time in the current study.

#### Academic Procrastination Scale

This scale (Solomon and Rothblum, 1984) consists of six areas of academic functioning (e.g., “writing for an exam”; “completing assignment”). Participants were asked to indicate on a five-point scale the degree to which they procrastinated on these tasks (1 = “never” to 5 = “always”). It has proved reliable and valid in the previous Chinese samples (Chen and Chang, 2016; Chen et al., 2016). Cronbach’s alpha was 0.93 in the current study.

#### Commitment to Learning Scale

Adolescents completed the Chinese version (Chen, 2017) of the Commitment to Learning Scale (Benson et al., 1998). It consists of seven items (e.g., “I am trying to learn new things,” and “I care about school”). Items were presented on a four-point scale ranging from 1 = “rarely” to 4 = “almost always.” The scale scores were computed by averaging the items. Cronbach’s alpha was 0.89 in the current sample.

## Results

### Descriptive Analyses

**Table 1** shows all means, standard deviations, and correlations. All four ecological assets were positively correlated with each other. In addition, all four ecological assets were negatively associated with academic procrastination, but positively associated with commitment to learning. Lastly, academic procrastination was negatively correlated with commitment to learning.

**Table 1:** Descriptive statistics of observed variables.

	1	2	3	4	5	6
(1) Support	–					
(2) Empowerment	0.73^∗∗∗^	–				
(3) Boundaries and expectations	0.75^∗∗∗^	0.78^∗∗∗^	–			
(4) Constructive use of time	0.49^∗∗∗^	0.48^∗∗∗^	0.45^∗∗∗^	–		
(5) Academic procrastination	-0.23^∗∗∗^	-0.26^∗∗∗^	-0.30^∗∗∗^	-0.13^∗∗^	–	
(6) Commitment to learning	0.60^∗∗∗^	0.63^∗∗∗^	0.69^∗∗∗^	0.38^∗∗∗^	-0.33^∗∗∗^	–
*N*	577	577	577	576	560	576
*M*	2.89	3.20	3.20	2.35	1.99	3.18
*SD*	0.63	0.61	0.64	0.80	1.05	0.63
*Skewness*	-0.15	-0.49	-0.57	0.47	1.18	-0.37
*Kurtosis*	-0.43	-0.20	-0.24	-0.43	0.82	-0.57

*^∗∗^*p* < 0.01; ^∗∗∗^*p* < 0.001*.

Since there were statistically significant positive correlations among some study variables, preliminary regression analyses were conducted to check multicollinearity effects using variance inflation factor (VIF). The VIF values ranged from 1.38 to 3.60, indicating that multicollinearity was not a problem.

### Main Analyses

The structural models, using Mplus 7.0 (Muthén and Muthén, 2012), were developed to test the hypotheses. First, the hypothesis that ecological assets were negatively correlated with academic procrastination was tested. We used four subscales (i.e., support, empowerment, boundaries and expectations, and constructive use of time) of the External Assets Scale to measure the ecological assets construct. The use of four subscales can provide more rigorous multi-indicator measurement of the ecological assets construct within the SEM framework. This multi-indicator approach is a psychometrical strength. The model fit the data well, χ^2^(5) = 14.35, *p* < 0.05, RMSEA = 0.06, CFI = 0.99, SRMR = 0.02. The result indicated that ecological assets was negatively correlated with academic procrastination (β = -0.30, *p* < 0.001), suggesting that participants who had more ecological assets had lower levels of academic procrastination.

Next, commitment to learning was added into the model to test the hypothesis that commitment to learning mediated the association between ecological assets and academic procrastination. The model fit the data well, χ^2^(8) = 22.06, *p* < 0.01, RMSEA = 0.06, CFI = 0.99, SRMR = 0.02. It indicated that ecological assets were positively related to commitment to learning (β = 0.74; *p* < 0.001), and commitment to learning was negatively related to academic procrastination (β = -0.24; *p* < 0.001). But the direct path between ecological assets and academic procrastination become statistically non-significant (β = -0.12; *p* = 0.07). This indicated that it was a full mediation model.

**FIGURE 1:**
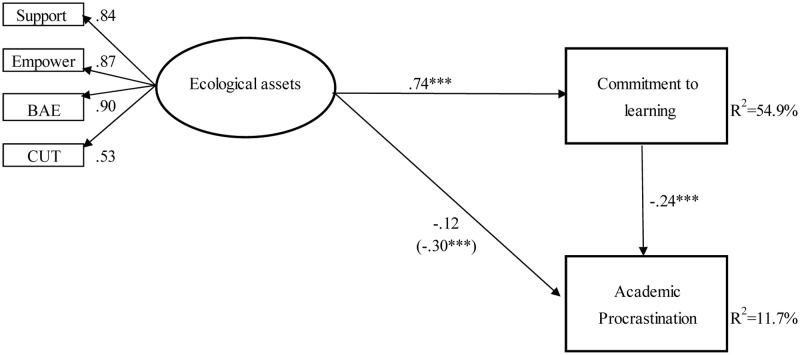
The model depicting the associations among ecological assets, commitment to learning and procrastination. Empower, empowerment; BAE, boundaries and expectations; CUT, constructive use of time. Number of missing data patterns = 4; ^∗∗∗^*p* < 0.001.

The significance of the mediating role of commitment to learning on the association between ecological assets and academic procrastination was tested using the Bootstrap estimation procedure. We generated 1000 bootstrapping samples from the original data set (*N* = 577) by random sampling. The standardized indirect effect of ecological assets on academic procrastination through commitment to learning was significant (point estimate = -0.18, *SE* = 0.05, *p* < 0.001, 95% CI = [-0.28; -0.08]). Therefore, the mediating effect of commitment to learning, proposed in the hypothesis, was supported (see **Figure 1**).

## Discussion

This study is one of the first to examine the relationships among ecological assets, commitment to learning, and academic procrastination among adolescents. Consistent with the hypothesis derived from the ecological model of resiliency, the results showed that ecological assets were negatively correlated with academic procrastination. It suggests that adolescents who have high levels of ecological assets may develop resilience traits that may discourage negative behavior such as academic procrastination. Therefore, this finding opens a novel avenue for understanding the origins of academic procrastination. In addition, the present finding adds to the existing PYD literature that a greater emphasis on building the ecological strengths among adolescents may decrease the likelihood of negative developmental outcomes.

In addition, the present study indicated that commitment to learning fully mediated the association between ecological assets and academic procrastination. It supports the ecological model of resiliency, whereby the intrapersonal assets may have a mediating role on the relationships between ecological assets and developmental outcomes. The current findings showing this full mediation may further contribute to the existing literature.

As suggested by some researchers (Scales et al., 2006; Benson, 2007), these ecological assets among adolescents may represent a potential resource for interventions and preventions against the academic procrastination. It is necessary for both families and schools to provide adolescents with social support, empowerment, boundaries and expectations, and constructive use of time. Building such ecological developmental assets deserves consideration as one of the strategies family and school can utilize to promote adolescents’ positive development. Thus, rather than attempting to find factors that cause them to academically procrastinate, it may be better to focus on addressing those factors that promote on-time academic behaviors in the first place.

The present findings are novel and important for several reasons. First, this research goes beyond prior studies of the environment—procrastination association by testing ecological assets at the level of latent variables rather than manifest variables. Second, this research provides new insights into the ecological factors on academic procrastination.

The present research has limitations. First, it is correlational in nature, limiting our ability to make inferences about the direction of effects among study variables. Future study should use a longitudinal design to confirm the proposed relationships. Second, the self-report measures may create method variance. Future studies will benefit from multiple sources of measures to assess study variables. Last, it is important to bear in mind that the findings apply to adolescents in China. There are age and cultural differences in procrastination (Steel, 2007; Klassen et al., 2009; Svartdal et al., 2016). For example, previous evidence indicated that Eastern Asian (e.g., Singaporean) adolescents reported higher levels of procrastination than Northern American (e.g., Canadian) adolescents (Klassen et al., 2009). It will be interesting to examine whether the associations among ecological assets, commitment to learning, and procrastination vary as a function of different cultural contexts. Despite these and other limitations, this study is one of the first to test the associations among ecological assets, commitment to learning, and academic procrastination among adolescents and, in doing so, further contributes to our understanding of the environmental origins of academic procrastination.

## Ethics Statement

This study was approved by the ethics committee of School of Social Development and Public Policy at Fudan University and University of Bergen Ethics Board. Informed consent was obtained from all individual participants included in the study.

## Author Contributions

B-BC developed the study concept and design, and collected data, B-BC and WH analyzed and interpreted the data, and drafted the manuscript.

## Conflict of Interest Statement

The authors declare that the research was conducted in the absence of any commercial or financial relationships that could be construed as a potential conflict of interest. The reviewer RS and handling Editor declared their shared affiliation.
